# Performance of Glass Wool Fibers in Asphalt Concrete Mixtures

**DOI:** 10.3390/ma13214699

**Published:** 2020-10-22

**Authors:** Agathon Honest Mrema, Si-Hyeon Noh, Oh-Sun Kwon, Jae-Jun Lee

**Affiliations:** 1 Department of Civil Engineering, Jeonbuk National University, Jeonju-si, Jeollabuk-do 54896, Korea; agathonmrema@gmail.com (A.H.M.); god02263@daum.net (S.-H.N.); 2 Principal Researcher, Korea Expressway Corporation Research Center, 208–96 Dongbu-daero 922beon-gil, Dongtan-myeon, Hwaseong-si, Gyeonggi-do 39660, Korea; pooh2461@ex.co.kr

**Keywords:** asphalt concrete, glass wool fibers, indirect tensile strength, Kim test, Marshall test, tensile strength ratio

## Abstract

Nowadays, in order to improve asphalt pavement performance and durability and reduce environmental pollution caused by hydrocarbon materials, many researchers are studying different ways of modifying asphalt concrete (AC) and finding alternative paving materials to extend the service life of pavements. One of the successful materials used in the modification of AC is fibers. Different types of fibers have been reinforced in AC mixtures and improvements have been observed. This research studies the performance of glass wool fibers reinforced in a dense-graded asphalt mixture. Generally, glass fibers are known to have excellent mechanical properties such as high tensile modulus, 100% elastic recovery and a very high tolerance to heat. Glass wool fibers are commonly used as a thermal insulation material. In this research, to evaluate the performance of glass wool fibers in AC, laboratory tests, the Marshall mix design test, indirect tensile strength (IDT), tensile strength ratio (TSR) and the Kim test were conducted to determine a proper mix design, tensile properties, moisture susceptibility, rutting and fatigue behaviors. Results show that the addition of glass wool fibers does affect the properties of AC mixtures. The use of glass wool fibers shows a positive consistence result, in which it improved the moisture susceptibility and rutting resistance of the AC. Additionally, results show that the addition of fiber increased tensile strength and toughness which indicates that fibers have a potential to resist distresses that occur on a surface of the road as a result of heavy traffic loading. The overall results show that the addition of glass wool fibers in AC mixtures is beneficial in improving properties of AC pavements.

## 1. Introduction

Asphalt concrete (AC), also known as hot-mix asphalt (HMA), is a combination of asphalt binder and aggregates mixed together at a high temperature. The AC is then placed and compacted on the road while still hot [1]. Asphalt pavement is the predominant pavement type in the world, and it is used for all types of applications from residential streets to expressways, from parking lots to harbor facilities and from bike paths to airport runways [1,2]. AC can be designed to form different types of mixtures depending on what the designer wants to achieve in terms of satisfactory performance over traffic and climate conditions of the designated region and durability of a pavement structure throughout the whole designed life expectancy [3,4].

After being applied to a road, AC mixtures tend to have many distresses such as cracking, rutting (permanent deformation), stripping (separation of aggregates from the AC mixture) and potholes. These distresses are caused by heavy traffic loading and harsh environment or weather conditions. The occurrence of distresses on road pavements may lead to a total failure of the pavement structure. Road engineers first and foremost have to ensure that they are avoiding all distresses at most, and to do so for the past few decades, engineers and material scientists have been involved in the process of modifying the traditional AC by adding other materials in it so as to improve its performance and durability and reduce costs while also trying to reduce environment pollution caused by the asphalt binder [5,6]. Different materials have been mixed together with asphalt binder and aggregates, but the most common ones are fibers and polymers [7]. Fibers, when added in asphalt mixture, have two main purposes which are to prevent drain down for gap- and open-graded asphalt mixtures and to increase strength and stability (reduce rutting) and improve resistance to cracking for dense-graded asphalt mixtures [7,8].

Cellulose (plant-based) and mineral fibers are used in AC to prevent drain down since they have high absorption capabilities [7,9,10]. Polypropylene, aramid, polyester and glass fibers have high tensile strength; therefore, they are used to add more strength to AC [7,11]. The process of adding fibers in either asphalt or cement concretes can be categorized into two categories: direct random inclusion (matrix), of which the outcome in AC is known as fiber-reinforced asphalt concrete, and the geo-synthetics family [11]. Polypropylene, cellulose and polyester fibers are the most prevalent fibers used in AC mixtures. Polypropylene fibers are preferred due to their low cost [12], strong bonding with asphalt, reduced cracking and shoving [7,13] and improved fatigue life [14]. Cellulose fibers are relatively inexpensive, widely available and stabilize the binder in gap- and open-graded stone matrix asphalt mixtures [15]. Polyester fibers resist cracking, rutting and potholes [7] and increase the stability and strength of the asphalt mixture [16].

This research studies a glass wool fiber reinforced in AC and its applicability. Glass wool is an insulating material made from fibers of glass arranged using a binder into a texture similar to wool [17]. Glass wool fibers have good flexibility, are easy to install, have high thermal resistance with low thermal conductivity (working temperature at about 450 °C) and are a fire-safe and environmentally friendly material [18]. Glass wool fibers are not commonly used in AC mixtures. Glass fibers, on the other hand, have been reinforced in AC mixture for years now [19]. Glass fibers have a high tensile modulus of about 60 GPa, low elongation of 3–4%, high elastic recovery, do not absorb water and have a high softening point (815 °C) [7]. When reinforced in AC mixture or cement concretes, glass fibers tend to increase the tensile strength and their compressive strength improves elastic recovery, and they increase the softening point of the asphalt binder [19,20,21,22].

The objectives of this research are to determine the effects of adding glass wool fibers in dense-graded AC in terms of strength properties, moisture susceptibility and fatigue properties, and determine the optimum fiber content to be used.

## 2. Materials and Experiment Method

### 2.1. Materials

**Figure 1:**
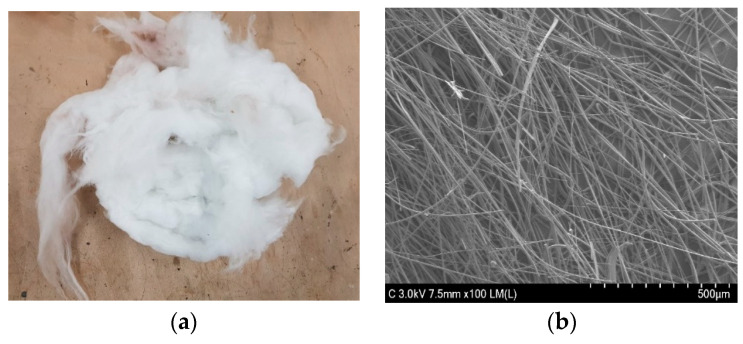
(**a**) Normal appearance of glass wool fibers and (**b**) SEM micrograph of glass wool fibers.

The materials used in this research include virgin asphalt binder with PG 64-20 produced by S-OIL company from Seoul Korea which was used in all AC samples and experiments. The properties of the binder used are given in Table 1 below. The dense-graded AC mixture used is known as Wearing Coarse-2 (WC-2), and it has a maximum aggregate size of 13 mm. WC-2 is commonly used in Korea as a surface asphalt pavement and it is highly water-resistant. Table 2 shows aggregate gradation, the required gradation limits and the aggregates’ sieve passing percentages used in this study, and as it is shown, aggregates used were all within the required limits. Figure 1 shows the normal appearance and an SEM micrograph of glass wool fibers used in this research. As shown in Figure 1, the glass wool fiber is white color. The thickness of the glass wool fiber is about 5~8 µm and it has a similar look to cotton candy. Table 3 shows the chemical composition of glass wool fiber.

### 2.2. Sample Preparations

In this research, glass wool fibers with a thickness of 5–8 µm were mixed in a dense-graded AC mixture together with asphalt binder and aggregates. One control sample with no fiber and another four samples with different percentages of glass wool fibers content (0.2%, 0.3%, 0.4% and 0.5% by weight of aggregates) were used. During mixing, fibers were mixed with aggregates first at a temperature of 150 °C for 1 min, and then asphalt binder was added. This method is known as a dry mixing method, and it is preferred over the wet mixing method because it allows the best fibers distribution in the mixture [19]. The already mixed hot AC samples were placed in molds and compacted, then cured for 24 h prior to testing.

### 2.3. Marshall Mix Design Method

The Marshall mix design is a common method used for designing dense-graded AC mixtures. It has two major features, namely density voids analysis and stability flow test [23]. In this research, the Marshall mix design test was done in accordance with the Korean standard for construction sector KS F 2337 [24]. Marshall specimens with a weight of 1200 g, width of 63.5 ± 1.27 mm and a diameter of 101.6 mm were compacted 75 times on both sides and then cured at room temperature for 24 h. For each set of fiber contents, there were at least 3 specimens. The Marshall mix design method was used to determine voids in mineral aggregates, air voids, voids filled with asphalt, bulk specific gravity Marshall stability and flow of AC mixtures with different dosages of glass wool fibers.

### 2.4. Indirect Tensile Strength

The indirect tensile strength (IDT) test is used to determine the tensile properties of the asphalt mixture; tensile properties include tensile strength, toughness and strain (displacement). Tensile properties play an important role in the performance of an AC mixture under fatigue, rutting and moisture susceptibility [25]. The IDT tests were conducted according to the Korean standard KS F 2382 [24], where a Marshall specimen with a weight of 1150 g and diameter of 101.6 mm, compacted 75 times on both sides, was loaded with compressive load at a constant rate of 51 mm/min acting parallel to and along the diametric plane of the specimen. The compressive load indirectly creates a tensile load on the horizontal direction of the sample and tensile failure occurs in a sample rather than a compressive failure [18]. Tensile strength values were obtained using the mathematical equation given bellow and the toughness was obtained from the dissipated energy during the material’s fracture process.

(1) $$S=\frac{2000\times P}{\pi tD}$$

where *S* = tensile strength, kPa; *P* = maximum load, N; *t* = specimen height before testing, mm; *D* = specimen diameter, mm.

### 2.5. Tensile Strength Ratio (TSR)

**Figure 2:**
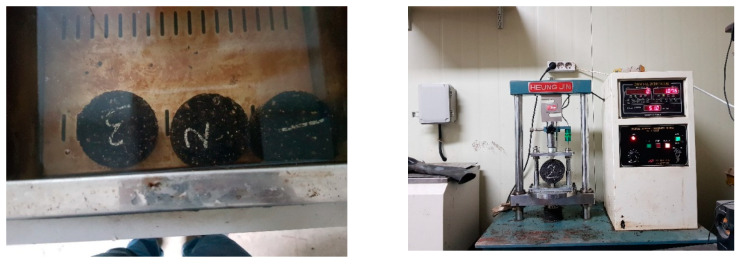
Tensile strength ratio test.

Tensile strength ratio (TSR) test is used to determine the moisture susceptibility of asphalt mixtures. It measures the workability of asphalt pavements in terms of performance and durability in the presence of moisture. The TSR test was carried out in accordance with the Korean standard KS F 2398 [24] as shown in Figure 2. The standard requires the TSR test to be done at a volume of air void of 7 ± 0.5%. Each set had 6 Marshall specimens, and after curing, 3 specimens were soaked in water at a temperature of 60 °C for 24 h and the other remaining specimens were kept dry at room temperature. TSR results are obtained as a ratio of the tensile strength values of conditioned samples which were soaked in water to the tensile strength values of unconditioned dry samples of the same mixture.

### 2.6. Kim Test for Deformation Strength

**Figure 3:**
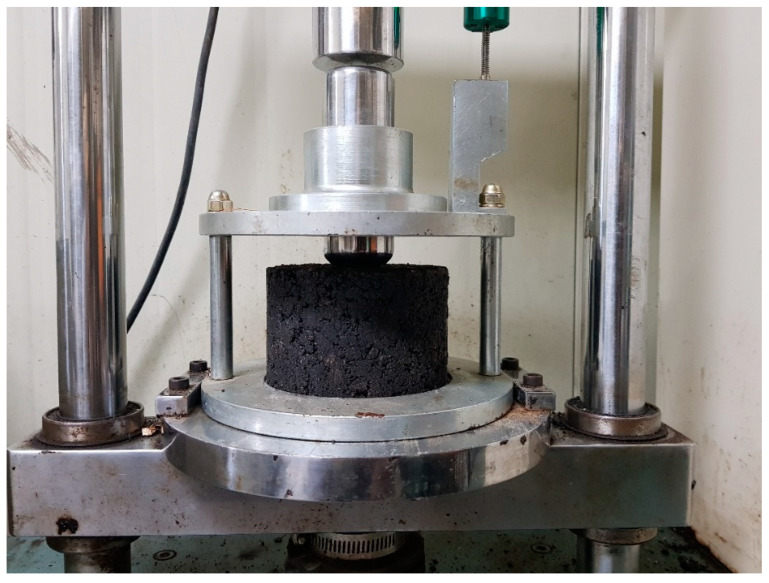
Kim test for deformation strength.

This is a new test recently developed in Korea for determining the rutting properties of AC mixtures. A round-edged loading head shown in Figure 3, which simulates almost the exact contact area between the tire and road surface, was applied instead of applying a load to the whole cross-sectional area. The resistance against the formation of a dimple (not flat) on the surface of the specimen is known as the deformation strength (*S_D_*). The deformation strength is a combination of compressive stress and shear stress. The Kim test showed good correlations with well-known rutting tests for dense-graded AC mixtures like wheel tracking [26,27]. The Kim test requires a normal Marshall specimen to be submerged in a water bath for 30 min at 60 °C, and then after a load is applied at a speed of 30 mm/min. The *S_D_* value is calculated as

(2) $$S_{D}=\frac{0.32P}{{\left[10+\sqrt{20y-y^{2}}\right]}^{2}}$$

where *S_D_* = deformation strength; *P* = maximum load; *y* = deformation.

### 2.7. Scanning Electron Microscopy (SEM)

In order to investigate the distribution of glass fiber wool in the asphalt mixture, scanning electron microscopy (SEM) was adopted in this study. The SEM device used was Hitachi, model SU8230 from Tokyo, Japan. This method was used to evaluate the dispersion of glass fiber wool and to ensure uniformity of the dispersion within the matrix of the asphalt mixture. Specimens were cut into small pieces of approximately (10 × 10 × 5) mm for testing, and the samples were then coated with a gold/palladium alloy to increase the stiffness at the surface and to obtain conductivity without affecting the observed surface morphology, in order to make them relatively more stable during the testing [28,29].

## 3. Results and Discussion

### 3.1. Optimum Asphalt Content (OAC)

**Figure 4:**
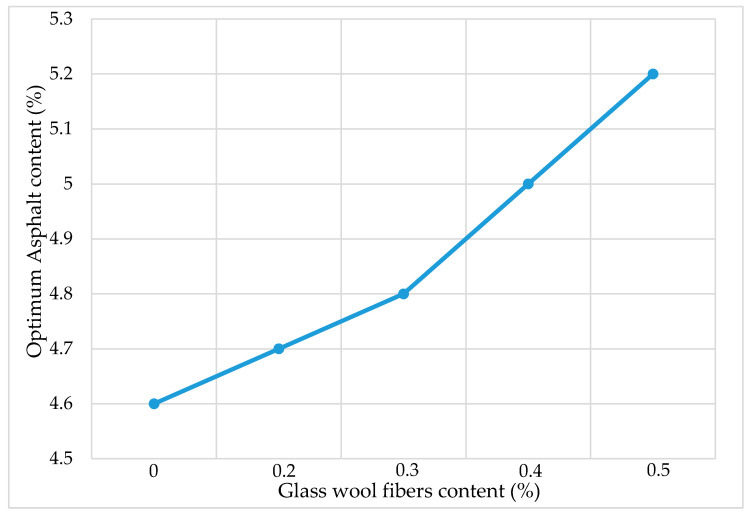
Optimum asphalt content (OAC).

**Figure 5:**
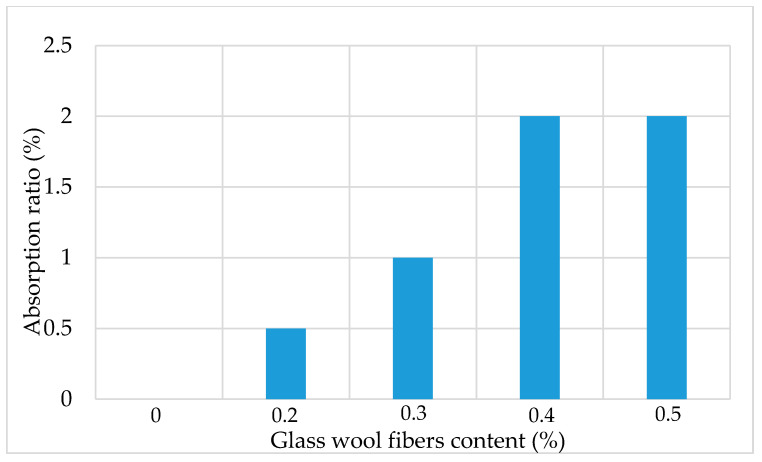
Glass wool fibers’ asphalt absorption ratio.

The optimum asphalt binder content for the fiber-reinforced AC mixture was determined using the Marshall mix design method. OAC is the amount of asphalt binder in terms of volume percentage, required to achieve the 4 ± 0.5% volume of air voids. The values of OAC were obtained through the interpolation of volume of air voids from three different asphalt contents at an increment of 0.5%. The OAC increases with the increase in fiber content, as shown in Figure 4. Generally, the fiber absorbed the asphalt binder in asphalt mixtures [30]. The glass wool fibers can absorb the asphalt binder, which leads to the addition of more binder. Figure 5 shows a trend of the asphalt binder absorption capabilities of glass wool fibers as a function of fiber content. The asphalt binder absorption ratio increased rapidly until 0.4% fiber content. It may be considered that the addition of fibers in AC is a complex process, and a more reasonable explanation needs to be investigated in the future.

### 3.2. Bulk Specific Gravity

**Figure 6:**
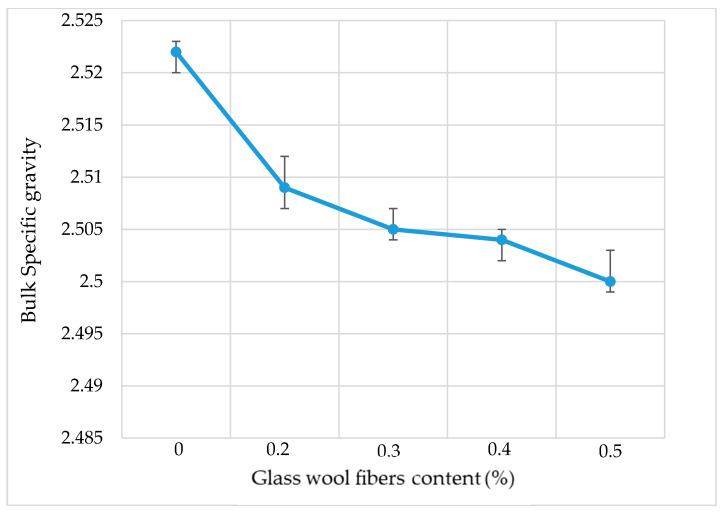
Bulk specific gravity.

During the Marshall mix design test, the bulk specific gravity of each dosage was measured and the results are given in Figure 6 below. The bulk specific gravity slightly decreases from 2.525 for the AC mixture with no fiber to 2.50 for the AC mixture with 0.5% of fiber content. The addition of fiber and the increase in optimum asphalt content led to a decrease in the aggregates volume used, which has the higher specific gravity, and hence the decrease in bulk specific gravity of the AC mixture.

### 3.3. Marshall Stability

**Figure 7:**
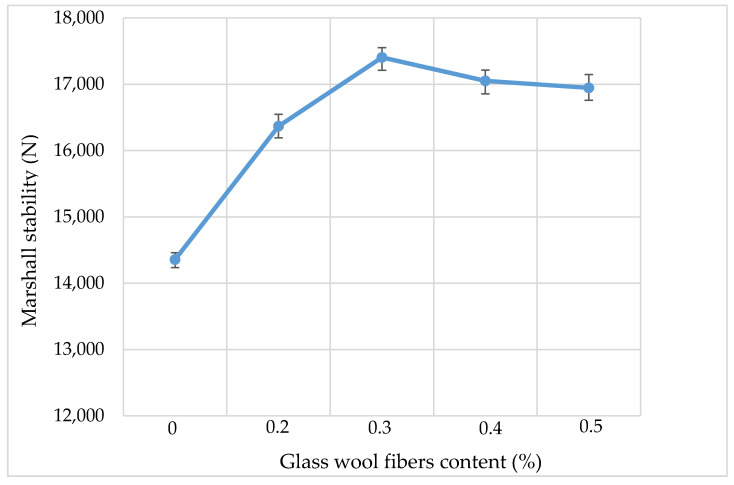
Marshall stability.

Figure 7 shows the Marshall stability results, and the measured results explain the resistance capabilities of a specimen related to distortion, displacement, rutting and shearing stresses under maximum loading. The results of each set were obtained from the Marshall stability of an asphalt content which correlated with the 4 ± 0.5% volume of the air void. Upon the addition of fibers, the Marshall stability increased and reached a peak when 0.3% of fiber content was added in the AC, and then it started to decrease. Glass fibers have high tensile strength which is transferred to the AC mixture and increases the Marshall stability.

### 3.4. Indirect Tensile Strength

**Figure 8:**
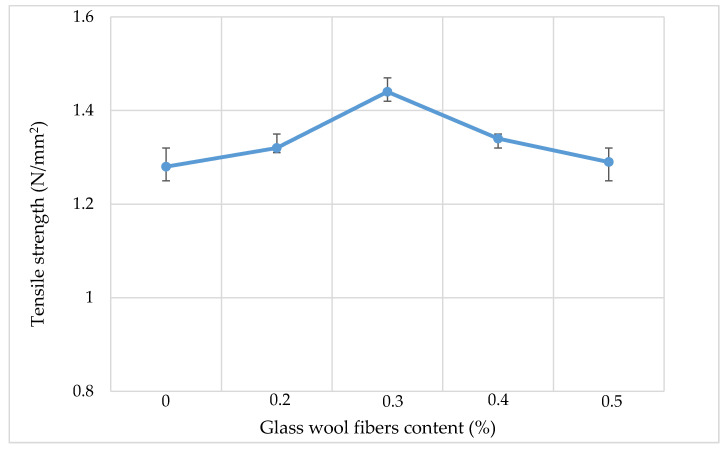
Tensile strength results.

**Figure 9:**
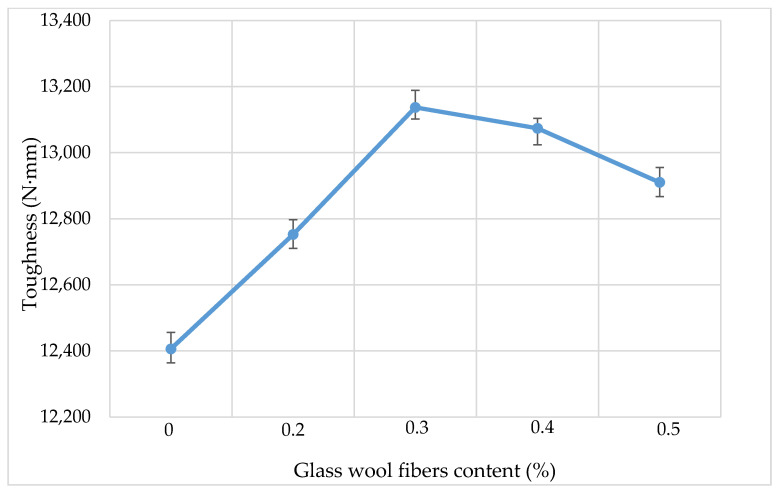
Toughness results.

Figure 8 and Figure 9 show the indirect tensile strength (IDT) results of asphalt mixtures containing different dosages of glass wool fibers. The results show that when fibers are added into the mixture, both the tensile strength and toughness of the AC mixture increase to a maximum value and then start to decrease. In asphalt pavements, tensile strength (stiffness) relates to the cracking properties of the pavement, with cracking being one of the primary asphalt pavement distress types along with rutting and fatigue. The resistance of AC to fatigue cracking which is caused by repeated or fluctuated stresses is dependent upon its tensile strength and extensibility characteristics. A higher tensile strength corresponds to a stronger crack resistance.

For the IDT test, each fiber content set had three specimens to be tested and results were obtained as an average. The Korean Asphalt standards guide for the AC mixture WC-2 given in Table 4 requires that tensile strength and toughness should be over 0.8 N/mm^2^ and 8000 Nmm, respectively. The tensile strength results show that tensile strength increased from 1.28 for a mixture without fibers to 1.44 N/mm^2^ for a mixture with 0.3% dosage of glass wool fibers, then for the higher dosage of fiber, the values of tensile strength decreased. Toughness results have the same trend as the tensile strength, where the maximum toughness value was observed at a mixture with 0.3% dosage of fibers, and for higher dosages, the toughness decreases. The decrease in tensile strength and toughness for higher dosages is caused by the large amount of fibers in a mixture which replace aggregates, while aggregates have higher tensile strength than fibers. Glass wool fibers, when added in an asphalt mixture, increase the strength of a bond between the asphalt and aggregates by firmly binding aggregate particles inside the matrix and preventing them from moving, which makes the mix stiffer [15].

### 3.5. Tensile Strength Ratio (TSR)

**Figure 10:**
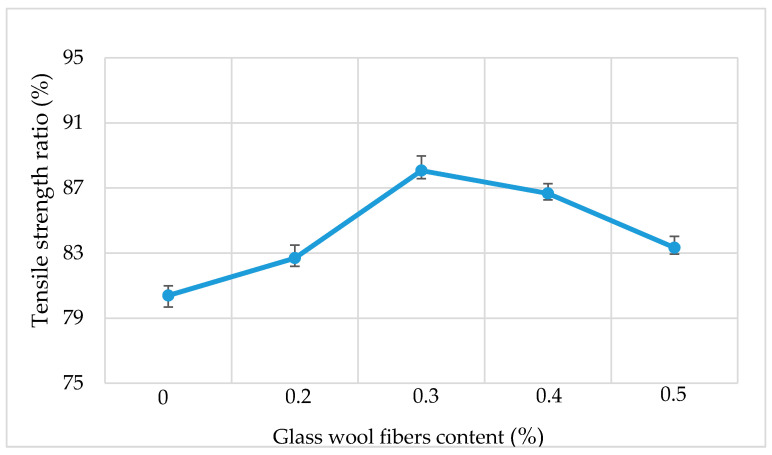
Tensile strength ratio results.

In order to estimate the moisture susceptibility of AC, the tensile strength ratio (TSR) test was conducted. Figure 10 shows the results of the tensile strength ratio (TSR) of asphalt mixtures for different dosages of fiber contents. As the amount of fiber dosage increases, the percentage of TSR also increases. The TSR increases up to a maximum value at a 0.3% dosage of fiber and then starts to decrease. A higher TSR value indicates that a mixture will have good resistance to moisture damages. The addition of fiber improves the moisture susceptibility of the AC mixture. The main reason behind this phenomenon is that when glass wool fibers interact with an asphalt mixture, this not only increases the bonding strength but also increases the thickness of the asphalt film which is beneficial for preventing moisture from entering the interface between the asphalt and aggregates.

### 3.6. Kim Test

**Figure 11:**
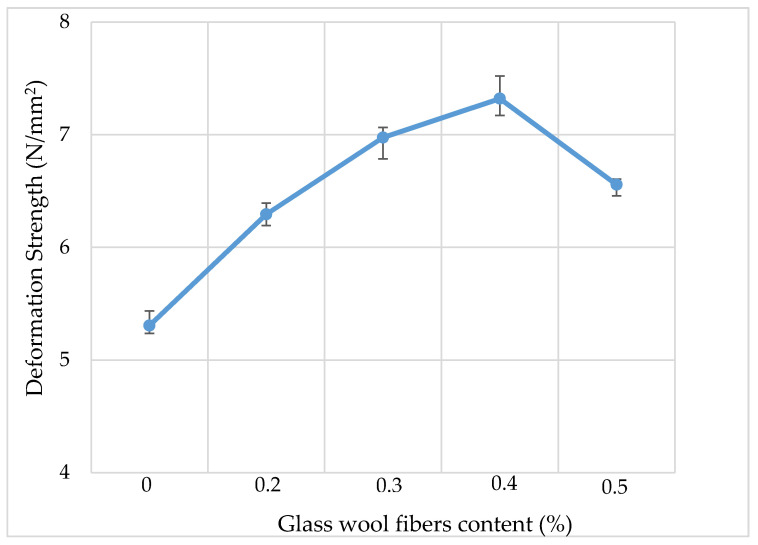
Kim test results.

Figure 11 shows the results of the Kim test for the deformation strength of AC mixtures with different dosages of glass wool fibers. The results show that the addition of fibers improves the high-temperature rutting properties of the AC. The *S_D_* values increased with the increased dosage of glass wool fibers, and they reached a maximum value at 7.32 N/mm^2^ when a dosage of 0.4% was added to the AC mixture, which is 25% more than the AC mixture without fibers. The *S_D_* values increased because glass wool fibers absorbed the asphalt binder and increased its viscosity, and therefore, due to the increased viscosity and bridging effects, the bonding strength between the asphalt and aggregates was increased.

### 3.7. Scanning Electronic Microscopy (SEM)

**Figure 12:**
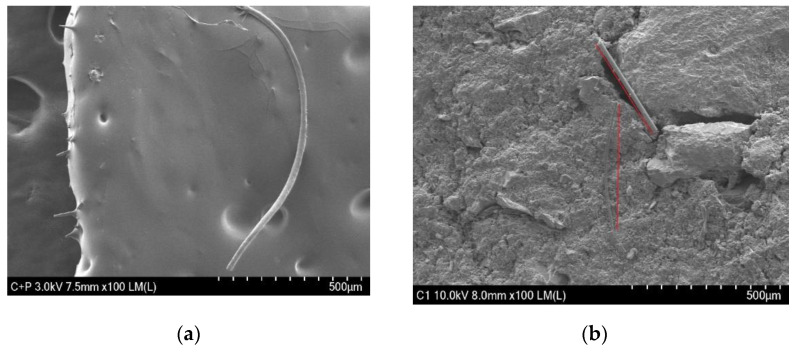
(**a**) Glass wool fibers mixed with asphalt binder and (**b**) asphalt concrete (AC) mixture with reinforced fibers.

**Figure 13:**
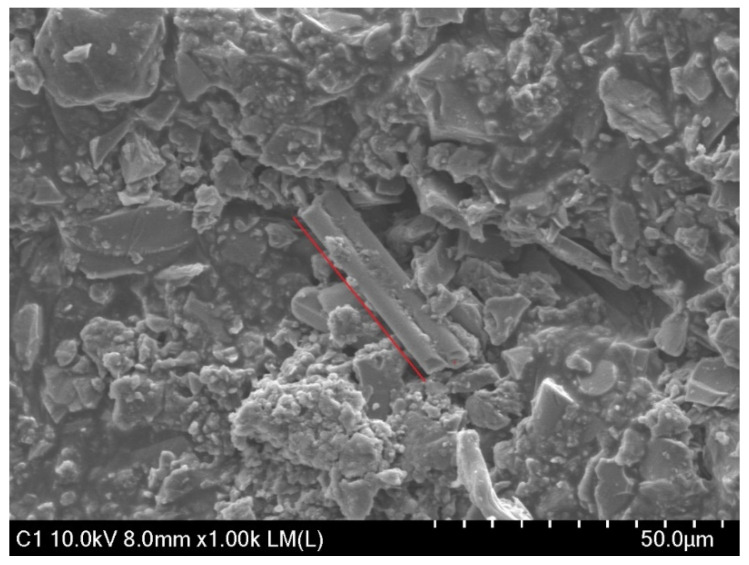
SEM micrograph of an AC mixture with fiber after indirect tensile strength (IDT) testing.

The SEM images of the fibers in an asphalt mixture are commonly used to show the dispersion of fibers in the mixture so as to understand the factors influencing the mechanical properties of the AC mixture. Images obtained by the use of scanning electron microscopy (SEM) device used Hitachi, model SU8230 from Tokyo, Japan are shown in the figures below. As shown in Figure 12a, the glass wool fibers were well immersed in the asphalt binder. The large portion of glass wool fibers was distributed in the asphalt mixture, and on the edges, there were spike-like fibers that emerged. The distribution of fibers in AC is mostly influenced by the size of fibers, thus when small-size fibers are well distributed in a mixture, this leads to an increase in the strength and stiffness of the mixture. In AC mixtures, glass wool fibers interact with the aggregates and binder, as shown in Figure 12b. Figure 13 shows a microstructure with a resolution of 50 µm of a destroyed part of an IDT sample after the test was conducted. Fibers interact with the aggregates and binder in the AC, and when tensile strength is applied to the AC, more tensile strength is needed to break the fibers.

## 4. Conclusions

This paper studied the performance of glass wool fibers in a dense-graded AC mixture. Glass wool fibers were added into the AC mixture together with an asphalt binder and aggregates. Laboratory tests, Marshall mix design, indirect tensile strength, tensile strength ratio and the Kim test were conducted to evaluate the effect of adding fibers to the AC. The following results were obtained based on laboratory investigations.

1. The addition of glass wool fibers to the AC mixture improves the performance of the AC in terms of tensile strength, toughness, rutting resistance, moisture susceptibility and fatigue.
2. The AC mixture with 0.3% of fiber content by weight of aggregates showed superior results over the other mixtures, where the results of indirect tensile strength, toughness, deformation strength and tensile strength ratio increased by 13%, 6%, 25% and 8%, respectively.
3. It was also observed that the addition of fibers leads to the addition of more asphalt binder. The addition of more binder enhances the durability of asphalt pavements.

Lastly, the authors suggest that more investigations should be done on the physical properties of glass wool fibers, fibers’ asphalt binder absorption capabilities and energy dispersive X-ray (EDX) analysis of the glass wool fibers. Additionally, since the glass wool fibers improved the tensile properties of AC and have high absorption capabilities, investigations should be done on reinforcing glass wool fibers in stone mastic asphalt mixtures.

## Figures and Tables

**Table 1:** Properties of virgin asphalt.

Property	Test Value	Standards
Penetration (25 °C, 100 g, 5 s) 0.1mm	63	ASTM D 5
Ductility(15 °C, 5 cm/min) (cm)	150	ASTM D 113
Softening (°C)	47.5	ASTM D 36
Flash Point (°C)	354	ASTM D 92
Density (15°C) (Kg/m^3^)	1.0410	ASTM D 70

**Table 2:** Gradation of asphalt mixture.

Sieve Size (mm)	25	20	13	10	5	2.5	0.6	0.3	0.15	0.08
Limits (%)	100	100	100–95	84–92	55–70	35–50	18–30	10–21	6–16	4–8
Passing (%)	100	100	96	85	56	36	19	12	7	5

**Table 3:** Chemical composition of glass wool fiber.

SiO_2_	Al_2_O_3_	Fe_2_O_3_	CaO	MgO	Na_2_O	K_2_O	SO_3_	B_2_O_3_	TiO_2_
66.9%	1.2%	0.2%	9.0%	1.9%	15.2%	0.3%	0.06%	5.0%	0.045

**Table 4:** Korean Asphalt standard for WC-2.

Test	WC-2	Etc
Marshall Stability (N)	Over 7500	Compaction 75 both sides
Air Void (%)	3–6	
Saturation Degree (%)	65–80	
TSR	Over 0.8	Air void 7 ± 0.5%
Tensile Strength (N/mm^2^)	Over 0.8	Compaction 75 both sides
Toughness (N × mm)	Over 8000	
Kim Test *S_D_* (N/mm^2^)	3.20	Air void 3–5%
